# Protocol for a Randomized Controlled Trial Testing the Efficacy of a Transdiagnostic Virtual Reality-Based Intervention for the Reduction of Unhealthy Lifestyles and Behaviors in the General Population

**DOI:** 10.3390/jcm12237470

**Published:** 2023-12-02

**Authors:** Valentina Gardini, Chiara Ruini, Eliana Tossani, Silvana Grandi, Elena Tomba

**Affiliations:** Department of Psychology, University of Bologna, 40127 Bologna, Italy; valentina.gardini8@unibo.it (V.G.); chiara.ruini@unibo.it (C.R.); eliana.tossani2@unibo.it (E.T.); silvana.grandi@unibo.it (S.G.)

**Keywords:** virtual reality, transdiagnostic factors, eating disorders, substance use disorder, alcohol use disorder

## Abstract

Virtual reality (VR) is a valuable tool for the treatment and prevention of psychiatric disorders and dysfunctional behaviors. Although VR software is mainly developed following a disorder-specific approach, this randomized controlled trial (RCT) will test the efficacy of a new transdiagnostic VR application (H.O.M.E. VR-based psychological intervention) in improving dysfunctional behaviors, three transdiagnostic factors concurrently (emotion regulation, experiential avoidance, and psychological flexibility), and stress. Three groups screened as at-risk for nicotine dependence, alcohol abuse, and eating disorders will be assigned to the H.O.M.E. VR intervention and compared to a waiting-list (WL) condition. Participants will be assessed before and after the H.O.M.E. intervention/WL and at the 3- and 6-month follow-ups in the levels of the displayed dysfunctional behavior, the three transdiagnostic factors, and stress. Changes in dysfunctional behaviors, transdiagnostic factors, and stress in each population VR group and differences in such improvements between each population of the VR and WL groups will be evaluated using mixed-model repeated measure analyses of variance. It is expected that, after the H.O.M.E. intervention and at follow-ups, participants will display improvements in physical and psychological health compared to controls. The H.O.M.E. protocol is expected to result in a cost-effective option to tackle cognitive–behavioral factors shared among several psychopathologies and dysfunctional behaviors.

## 1. Introduction

The term “Virtual Reality” (VR) refers to an array of technologies that allow individuals to experience and interact with computer-generated three-dimensional environments and objects through a head-mounted display [1]. The high levels of immersivity and realism offered by VR make individuals experience a sense of “emotional presence”, which is comparable to reality in inducing emotional responses [2,3]. Because of this, VR has been used in clinical psychological contexts as an instrument to offer patients systematic and controlled exposure therapy without the complications of in vivo exposure [4]. VR is also capable of improving existing cognitive-behavioral treatments (CBT) and prevention protocols for several psychiatric disorders, especially in patients with anxiety, psychosis, substance-related, and eating disorders (EDs) [5,6]. Although VR software and protocols have been developed mainly following a disorder-specific approach [6,7], VR programs capable of being administered transdiagnostically across several psychiatric diagnoses have also been recently designed for the improvement in transdiagnostic aspects, particularly emotion regulation strategies [8].

According to the transdiagnostic approach, many psychological disorders and comorbid psychiatric diagnoses are maintained or caused by similar cognitive and/or behavioral processes [9,10,11], defined as transdiagnostic factors. While research has not reached a consensus regarding which transdiagnostic factors should be mainly implicated in the development of psychological disorders, the assumption that certain shared cognitive and behavioral processes maintain or lead to the onset of many psychological disorders has been supported in the literature [11,12]. Within the third wave of the cognitive behavioral theory of mental disorders [13], clinical studies showed that several behaviors that might be detrimental to individuals’ health (such as heavy drinking, heavy smoking, or dysfunctional eating behaviors) are related to high levels of psychological inflexibility [14,15,16], emotion dysregulation [17,18,19], and experiential avoidance [20,21]. These transdiagnostic factors have also been found to correlate to higher stress levels [22,23,24]. Indeed, some transdiagnostic VR software protocols have been developed for the improvement in emotion regulation (ER) strategies [8] and were found capable of reducing unhealthy behaviors and lifestyles (e.g., heavy smoking, heavy drinking, or dysfunctional eating behaviors) in the general population [5,6,25,26,27,28,29,30]. However, no transdiagnostic VR software or intervention has been designed yet to tackle more than one unhealthy behavior within the same software. Moreover, the beneficial effects of VR-based interventions, including the improvement in experiential avoidance and psychological flexibility, have not been proven yet. In particular, no VR software has been designed to concurrently tackle these third-wave cognitive–behavioral transdiagnostic factors and multiple unhealthy behaviors and lifestyles.

To fill this gap in the literature, researchers and clinicians (clinical psychologists and psychotherapists E.Tom., C.R., and E.Tos. and psychiatrist and psychotherapist S.G.) from the Department of Psychology of the University of Bologna designed a transdiagnostic VR software and intervention protocol (H.O.M.E.—How to Observe and Modify Emotions) to improve emotion regulation, experiential avoidance, and psychological flexibility in order to make them suitable for several clinical and non-clinical populations. The H.O.M.E. intervention was designed to be a six-session intervention protocol focusing on the assessment of emotions elicited by trigger cues present in the virtual environment (e.g., comfort foods, cigarettes, alcohol, etc.) and the tackling of these emotions through CBT-based exercises to enhance the aforementioned transdiagnostic factors.

In particular, this study presents the research protocol for a randomized controlled trial designed to evaluate the effects of H.O.M.E. VR-based intervention in ameliorating the frequency and severity of unhealthy behaviors (i.e., heavy smoking, heavy drinking, and dysfunctional eating behaviors) and three transdiagnostic factors (emotion regulation, experiential avoidance, and psychological flexibility) levels in individuals of the general population that are considered at risk for nicotine dependence, alcohol abuse, or eating disorders. These experimental groups will be compared to a waiting-list condition consisting of a 6-week duration with weekly phone check-ups. The hypotheses are that H.O.M.E. will prove to be effective in reducing the frequency and severity of unhealthy behaviors as well as improving the transdiagnostic factors, even when compared to the waiting-list condition.

## 2. Materials and Methods

### 2.1. Design

The present study is a randomized controlled trial (RCT) protocol involving individuals from the general population presenting three different types of unhealthy lifestyles and behaviors (i.e., people who engage in heavy smoking, heavy drinking, or report dysfunctional eating behaviors), each one randomized into two treatment arms: an intervention group that will receive the H.O.M.E. VR-based intervention and a control group assigned to a waiting-list condition. The RCT follows the guidelines of the Standard Protocol Items: Recommendations for Interventional Trials (SPIRIT) [31] and the Consolidated Standards of Reporting Trials (CONSORT) [32] and was approved by the ethical committee of the Department of Psychology, University of Bologna (Protocol N. 314877, 14 December 2021).

### 2.2. Participants and Sample Size

Individuals from the general population (age range: 18–60 years) exhibiting unhealthy lifestyles and behaviors (i.e., heavy smoking, heavy drinking, and dysfunctional eating behaviors) and are at risk for nicotine dependence, alcohol abuse, or eating disorders will be recruited via social media by filling an online battery of self-report psychometric questionnaires (administered via the Qualtrics online platform).

The inclusion criteria will include the following: (a) being between 18 and 60 years of age; (b) having no prior diagnosis according to the DSM-5-TR [33] diagnostic criteria; (c) displaying one of the unhealthy lifestyles and behaviors mentioned above according to the relative screening questionnaires (see Screening measures below).

The exclusion criteria will include the following: (a) lack of capacity to consent for research; (b) current or former diagnosis of psychiatric disorders according to the DSM-5-TR diagnostic criteria; (c) using medical devices (e.g., pacemaker and hearing aids) or having a medical condition (e.g., vertigo and vision impairments) that may interfere with VR technologies; (d) having concurrent physical health conditions that might better explain the presence of these unhealthy behaviors.

Participants will be enrolled until reaching a number of around 64 individuals for each of these three separate samples: (1) individuals who engage in heavy smoking, (2) individuals who engage in heavy drinking, and (3) individuals who report dysfunctional eating behaviors. The sample size was calculated using G*Power [34] and considering our main statistical analyses (repeated measure ANOVAs with a medium effect size of 0.25, power of 0.80, two groups, and four time measurements), with an addition of an estimated 30% drop-out rate.

### 2.3. Procedure

In the screening phase, participants will fill in online (via the Qualtrics platform) a non-psychometric self-report questionnaire and several self-report psychometric screening questionnaires for the detection of three unhealthy lifestyles and behaviors: heavy smoking, heavy drinking, and dysfunctional eating behaviors (see Screening measures). Individuals interested in taking part in the research will be informed of the aims and characteristics of the study. If they agree to participate, they will be asked to sign an informed consent online on Qualtrics. Participants who provide written informed consent will be screened, and if suitable to participate, will be subsequently contacted via e-mail or smartphone within 7 days and invited to take part in the experimental phase of the study.

In the experimental phase, participants in each of the three samples will be randomized to the intervention (VR) or control (waiting-list) condition following a “block randomization” method [35]. Before randomization, participants will undergo a brief clinical interview based on the DSM-5-TR criteria conducted by a charted clinical psychologist researcher to exclude the presence of DSM-5-TR psychiatric disorder diagnoses. Participants in the VR group will undergo six 30 min sessions of the VR-based intervention administered using the software H.O.M.E. in the presence of a clinical psychologist (the intervention protocol is illustrated below). Controls (waiting-list group) will not receive any intervention during the study, but they will receive minimal attention from researchers through phone check-up sessions to monitor their symptoms and general well-being. They will also be offered to receive the VR-based intervention after concluding the study.

To test whether changes will be maintained over time, both groups will be re-contacted for follow-ups after 3 and 6 months.

Immediately before the first session of the intervention phase (T0), at the conclusion of the intervention phase (T1), and at the 3 (T2) and 6-month follow-ups (T3), participants of all groups will be asked to complete a series of psychometric self-report questionnaires online (using the Qualtrics platform) (see Outcome measures).

**Figure 1:**
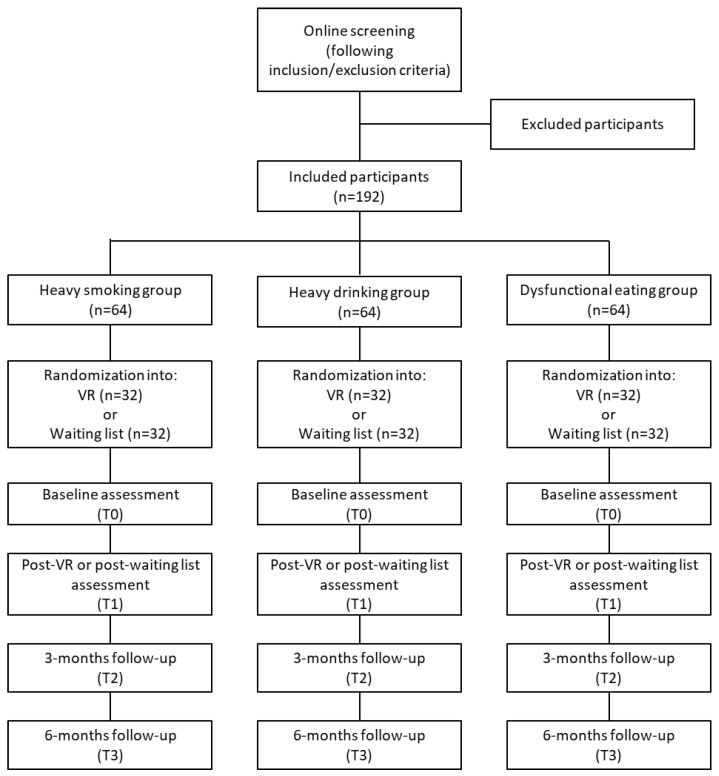
Flow chart for the randomized controlled trial (RCT).

More details about the allocation of participants and the RCT design are shown in Figure 1.

### 2.4. Interventions

#### 2.4.1. VR Software: H.O.M.E. (How to Observe and Modify Emotions)

**Figure 2:**
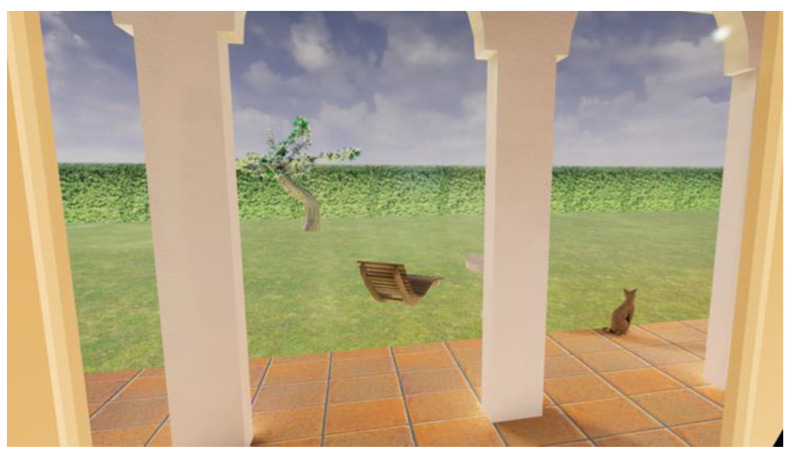

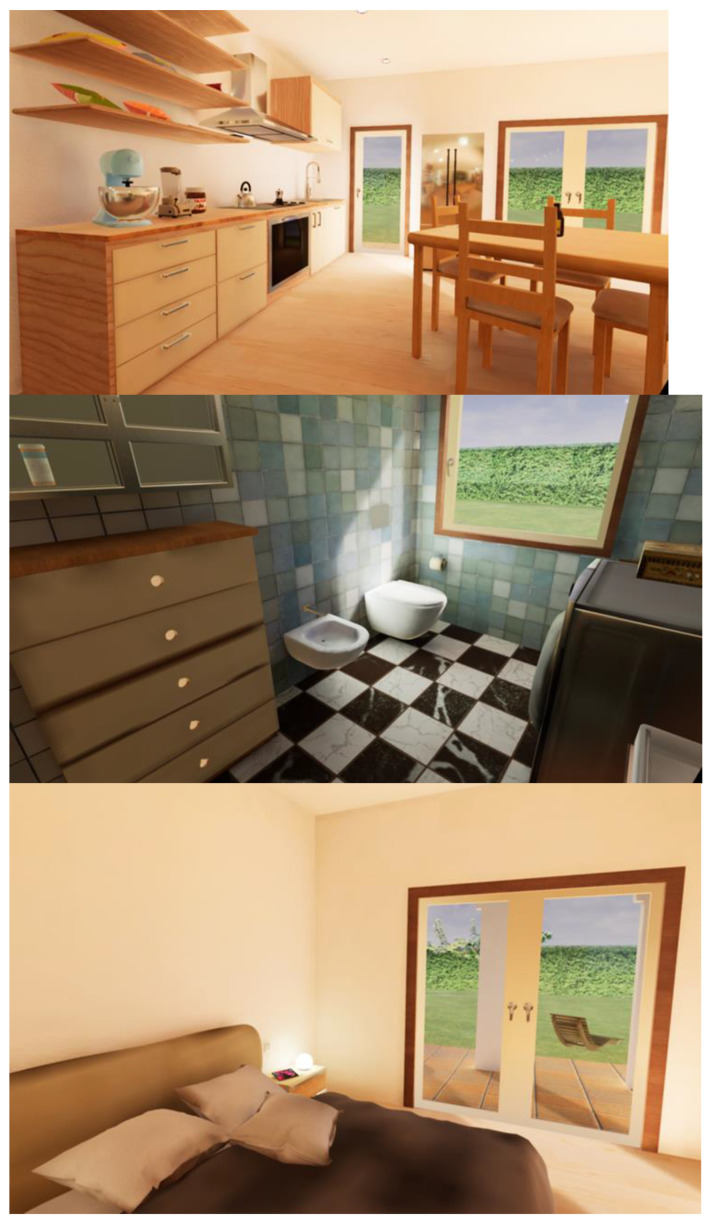

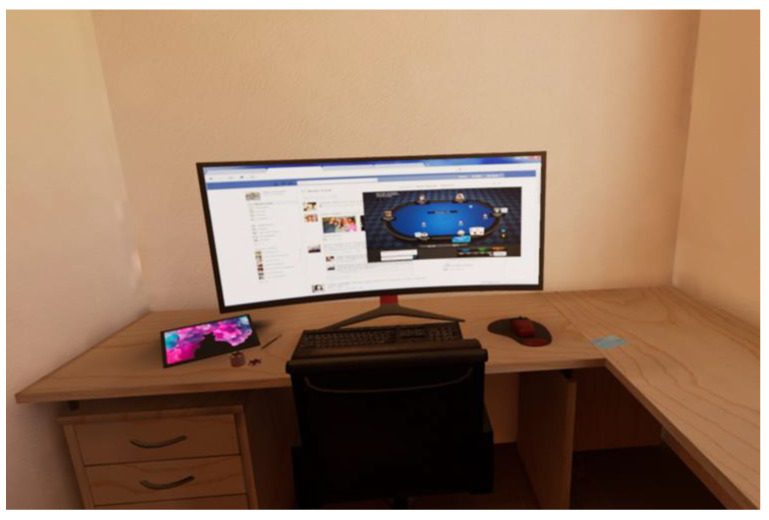
Locations present in the H.O.M.E. VR software (vr. 1).

**Figure 3:**
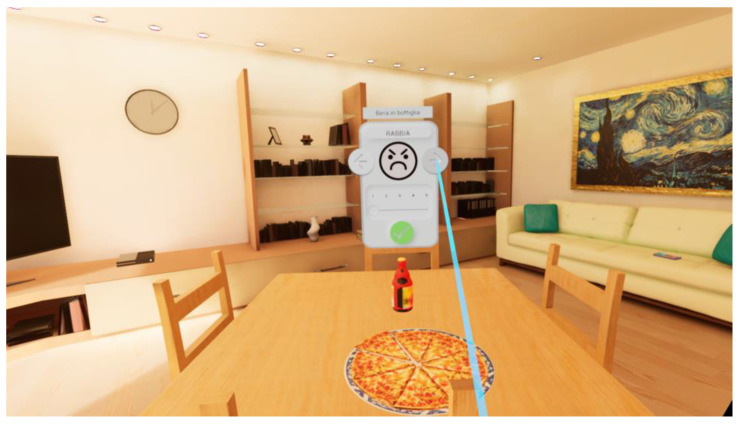
The emotional assessment feature of the H.O.M.E. VR software.

**Figure 4:**
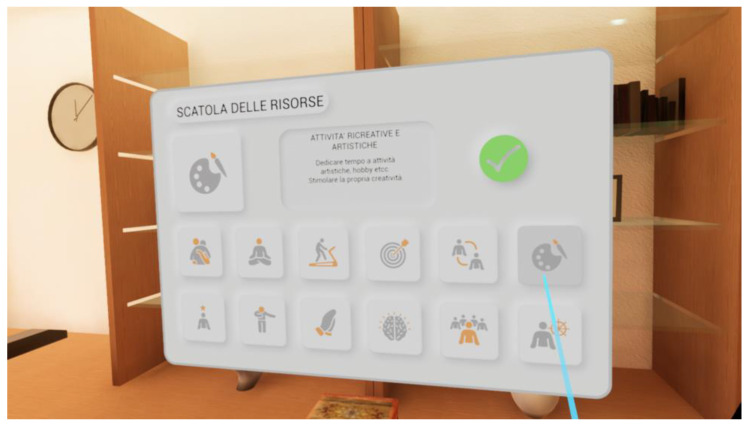
Box of psychological resources (intervention feature) of the H.O.M.E. VR software.

H.O.M.E. (How to Observe and Modify Emotions) is a transdiagnostic VR software (vrs.1) developed by a group of clinical psychology researchers (clinical psychologists and psychotherapists E.T., C.R., and E.T. and psychiatrist and psychotherapist S.G.) from the Department of Psychology at the University of Bologna. H.O.M.E. can be used by connecting a computer to any VR headset (in this case, an HTC Vive headset will be used for the study). The software offers users the opportunity to move around a virtual environment consisting of a house with four rooms (i.e., kitchen/living room, bathroom, bedroom, and a study; Figure 2) and a garden and to interact with objects representing likely relevant cues for specific at-risk behaviors (e.g., comfort foods for people with eating-related issues; alcohol, pills, and cigarettes for users with substance use disorders; a computer with a gambling interface for those with gambling addictions; videogames and a smartphone for individuals with technology-related addictions). The software includes two separate but interrelated virtual features: an assessment virtual feature (Figure 3) in which users can attribute to each object the emotion they associate with it and its intensity; and an intervention virtual feature (Figure 4) based on the cognitive-behavioral model in which users can access a box containing “psychological resources”, including pictures and descriptions of twelve emotional, social, and behavioral resources that can be used to deal with the emotions previously attributed to the following objects: (1) forgiveness; (2) awareness; (3) physical activity; (4) life purposes; (5) interpersonal relationships; (6) recreational activities (e.g., hobbies); (7) self-esteem; (8) gratitude; (9) transcendence and spirituality; (10) wisdom; (11) autonomy-assertiveness; and (12) self-control. These resources have been derived from theoretical models at the base of third-wave cognitive behavioral therapies (e.g., mindfulness-based therapy, acceptance and commitment therapy, dialectical behavioral therapy, positive psychotherapy, etc.), such as the psychological well-being model of Carol Ryff [36] and the value in action (VIA) classification of the character’s strengths and virtues model [37]. The software is meant to be transdiagnostic since it is designed to tackle the following transdiagnostic factors: emotion regulation (ER) strategies, psychological flexibility, and experiential avoidance, which are meant to be used in the prevention and treatment of several psychiatric disorders (e.g., EDs, substance-use disorders, addictions, etc.) and severe levels of stress under the instructions of a clinical psychologist. The need for a clinical psychologist when using H.O.M.E. represents another innovative aspect of this software, as it allows the inclusion of clinical judgment when using this technological tool in a VR-based intervention. According to the aims of this specific research project, H.O.M.E. will be used to deliver a VR-based assessment and intervention in individuals from the general population who exhibit unhealthy lifestyles and behaviors (i.e., heavy smoking, heavy drinking, and dysfunctional eating behaviors) and are at risk for nicotine dependence, alcohol abuse, or eating disorders.

##### Virtual Reality Devices

The devices used to deliver the VR intervention will include a computer and an HTC Vive VR headset. The HTC Vive headset will be connected to a computer in order to access the VR software used for the study: the H.O.M.E. (How to Observe and Modify Emotions) software (described below). Participants will be able to navigate the virtual environment using the HTC Vive headset and controllers. During the intervention session, the clinical psychologist will also be able to see the participant’s actions in the virtual environment and, therefore, they will be able to give instructions to the participants.

##### VR-Based Intervention Group

The proposed VR-based intervention will consist of six weekly sessions of approximately 30–50 min. The intervention will take place at the VR laboratory of the Department of Psychology of the University of Bologna. The intervention protocol will be delivered by a clinical psychologist (author V.G.).

The six VR-based intervention sessions will have different aims and will be divided as follows:

(1) Session 1 will focus on gathering information about the participants and instructing them on the use of virtual reality interfaces. During this session, the participants will have the time to familiarize themselves with the H.O.M.E. software VR environment, and the clinician will verify if any motion sickness symptoms or difficulties in using the VR device are reported by participants.
(2) Session 2 will focus on the interaction with the objects in the virtual HOME environment that are the most relevant for the participant (i.e., foods, drinks, electronic devices, cigarettes, etc.). During this session, the participant will be asked to attribute to each relevant object the emotions they associate with it and its intensity (this will be performed via the assessment phase of the H.O.M.E. software). CBT-based homework (in particular, filling in an Antecedents, Behavior, Consequences-ABC worksheet throughout the subsequent week) will be given to the participant at the end of the session.
(3) Session 3 will focus on recreating critical situations that the patient wrote in the ABC worksheet in the H.O.M.E. software and further discussing the relevant objects and elicited emotions.
(4) Session 4 will focus on illustrating to the participants all the psychological/behavioral resources present in the “box of resources”. The participants will then be asked (with help from the clinical psychologist, if needed) to select the resource they think will be the most beneficial to overcome the distressing emotions previously attributed to the critical objects (this is performed using the intervention phase of the H.O.M.E. software). The clinician will also discuss with participants how to use this resource in the real world, and the participants will be asked to apply it in everyday life during the following week as homework.
(5) Session 5 will focus on discussing the difficulties participants might have experienced when trying to use the resource in their everyday life, and if needed, additional exercises will be conducted in the virtual environment to better use the resource. Alternatively, the participant might be asked to select an additional resource they would like to explore.
(6) Session 6 will focus on re-evaluating the objects the participant previously selected as critical and evaluated in Session 2 to assess possible changes in the intensity and type of emotions attributed to them after using the psychological resources to deal with them in a real-life context.

To test whether changes will be maintained over time, participants will be re-contacted for follow-ups after 3 and 6 months.

#### 2.4.2. Control (Waiting-List) Group

The control group in this study consists of a 6-week waiting-list condition. Participants in this group will not receive VR-based interventions or any other interventions during the study. However, minimal attention from researchers will be paid to controls via phone check-up sessions to monitor their dysfunctional behaviors and general well-being. The phone check-ups will also be carried out by a charted clinical psychologist (author E.Tom.). Controls will also be offered to receive the VR-based intervention after six weeks.

Participants in the WL condition will also be recontacted after 3 and 6 months for follow-ups to assess whether changes will be maintained over time.

### 2.5. Measures

Participants in both the control/waiting-list and VR/experimental groups will fill in the same screening and outcome measures.

#### 2.5.1. Screening Measures

All participants who will provide written informed consent and meet the criteria to be included in the experimental part of the study will be asked to fill in the following questionnaires:

− A non-psychometric self-report questionnaire to collect socio-demographic (age, marital status, educational level, and occupational status) and clinical data (body mass index (BMI), former DSM-5-TR diagnosis, and clinical conditions and/or use of medical devices interfering with VR) and to investigate the participants’ familiarity with the use of a computer or technological devices (i.e., computer and videogames) in their everyday life.
− The Alcohol Use Disorders Identification Test (AUDIT) [38] is a 10-item psychometric screening tool developed by the World Health Organization (WHO) to assess alcohol consumption, drinking behaviors, and alcohol-related problems. AUDIT scores higher than five indicate a potentially harmful pattern of drinking, with a sensitivity of 0.84 and a specificity of 0.90 in the Italian population [39].
− The Fagerström Test for Nicotine Dependence (FTND) [40,41] is a 6-item self-report psychometric instrument for assessing the intensity of physical addiction to nicotine in terms of the quantity of cigarette consumption, compulsion to use, and dependence. Scores between three and four at the FTND indicate a low-to-moderate dependence, scores between five and seven indicate a moderate dependence, and scores higher than eight indicate a high dependence. The Italian version of the FTND showed low internal consistency reliability, with a Cronbach’s alpha of 0.55 in the combined sample (=0.59 among men and =0.50 among women) [41].
− The Eating Disorders Examination Questionnaire (EDE-Q 6.0) [42,43] is a 28-item psychometric self-report questionnaire to evaluate their levels of ED psychopathology. The EDE-Q focuses on the past 28 days and produces a global score as well as four subscales: Eating Concern, Shape Concern, Weight Concern, and Restraint. The EDE-Q also includes items measuring the frequency of core ED behaviors, such as binge eating and compensatory behaviors. Higher scores are indicative of higher eating pathology, and scores between 1.56 and 3.91 are found to be associated with ED risk in the GP [44]. The EDE-Q demonstrated satisfactory concurrent validity [42,43] and acceptable internal consistency (Cronbach’s α ranging from 0.70 to 0.83 for the subscale and ≥0.90 for the global score) [45].

Participants will be selected to participate in the intervention phase if they score at least 5 on the AUDIT, 7 on the FTND, or 1.56 on the EDE-Q and assigned to the respective group, according to the displayed dysfunctional behavior.

#### 2.5.2. Outcome Measures

Changes in the levels of endorsed unhealthy behaviors will be measured by administering the FTND, AUDIT, SCOFF, and EDE-Q at the beginning (T0) and end (T1) of the VR intervention to the participants of the relative sample, as well as at the 3- (T2) and 6-month (T3) follow-up.

The following instruments will instead be administered to participants of all samples at T0, T1, T2, and T3 to evaluate their levels in the three considered transdiagnostic factors (i.e., emotion regulation, experiential avoidance, and psychological flexibility) as well as participants’ levels of stress:

− The Difficulties in Emotion Regulation Scale-brief version (DERS-16) [46], which is a 16-item self-report psychometric questionnaire evaluating six ER strategies: 1. non-acceptance of emotional responses, 2. difficulty in engaging in goal-directed behaviors, 3. impulse control difficulties, 4. lack of emotional awareness, 5. limited access to ER strategies, and 6. a lack of emotional clarity. DERS-16 reported good test–retest reliability [46] and excellent internal consistency (Cronbach’s alphas between 0.87 and 0.96) [47]. The DERS-16 scores were also found to strongly correlate with levels of psychological distress in clinical and non-clinical populations [46,48,49,50].
− The Acceptance and Action Questionnaire-II (AAQ-II) [51,52] is a 7-item self-report psychometric questionnaire assessing psychological flexibility. The Italian version of the AAQ-II proved to be a reliable and valid measure of psychological inflexibility, with high internal consistency (0.83) and modest test–retest reliability over a 12-month period (0.61) [51].
− The Experiential Avoidance Scale (MPFI-EA) from the Multidimensional Psychological Flexibility Inventory (MPFI) [53,54], which is a 5-item self-report psychometric scale evaluating experiential avoidance taken from the MPFI, and a 60-item self-report questionnaire assessing global psychological flexibility and inflexibility processes. MPFI-EA showed high reliability in the Italian version (Cronbach’s alpha = 0.91) [53].
− The Depression, Anxiety, and Stress Scale—21 (DASS-21) [55,56] is a 21-item self-report psychometric questionnaire designed to measure the emotional states of depression, anxiety, and stress. Each of the three DASS-21 scales contains seven items that are divided into subscales. In particular, the stress scale is sensitive to the levels of chronic non-specific arousal. It assesses difficulty relaxing, nervous arousal, being easily upset/agitated, irritable/over-reactive, and impatience. Scores between 19 and 25 on the DASS-21 stress scale indicate moderate levels of severe levels of stress, scores between 26 and 33 indicate severe stress, and scores higher than 34 indicate extremely severe levels of stress. The Italian version of the DASS-21 demonstrated good internal reliability, with Cronbach’s alpha of 0.90 in the community sample (Cronbach’s alpha = 0.85 for the stress scale) [55].

### 2.6. Data Analyses

Data analyses will be performed using SPSS Statistic vrs.28. Data analyses will be performed separately for the three samples that are included in the study (i.e., individuals who engage in heavy smoking, heavy drinking, and dysfunctional eating behaviors).

Descriptive statistics will be run to analyze socio-demographics (age, marital status, educational level, and occupational status), clinical characteristics (BMI, former DSM-5-TR diagnosis, clinical conditions, and/or the use of medical devices interfering with VR), and mean FTND, AUDIT, SCOFF, and EDE-Q scores in the relative groups. The mean DERS-16, AAQ-II, MPFI-EA, and DASS-21-stress scale scores will also be calculated for all samples separately.

To test pre-intervention differences between the experimental and control groups (waiting-list condition) in each sample, Chi-squares will be performed on categorical variables, whereas ANOVAs will be performed on continuous variables.

To test the effects of the VR-based intervention, changes over time in the relevant unhealthy behavior (FTND, AUDIT, or SCOFF and EDE-Q scores), in transdiagnostic factors (DERS-16, AAQ-II, and MPFI-EA scores), and in stress levels (DASS-21-stress scale scores) will be assessed in the experimental group of the samples using a priori contrast analysis. In particular, we plan to compare each assessment time (T0, T1, T2, and T3) against each other. The percentage of drop-out cases will be calculated.

To test the efficacy of the VR-based intervention compared to the control (waiting-list) condition, changes between the experimental and control (waiting-list) groups of the samples in the relevant unhealthy behavior (FTND, AUDIT, and EDE-Q scores), in transdiagnostic factors (DERS-16, AAQ-II, and MPFI-EA scores), and in stress levels (DASS-21-stress scale scores) will be tested by performing repeated measure ANOVAs using time (T0, T1, T2, and T3) as a within-subject factor and group assignment (experimental/VR vs. control) as a between-subject factor. To control for Type 1 error in within-group mean contrasts, the Bonferroni correction will be applied.

Moreover, to also take into consideration the possible multivariate nature of the involved variables and the association between dysfunctional behaviors, transdiagnostic factors, and stress, we will perform repeated measures MANOVAs using time (T0, T1, T2, and T3) as a within-subject factor, group assignment (experimental/VR vs. control) as a between-subject factor, and questionnaire scores (one between FTND, AUDIT, or EDE-Q scores depending on the sample, as well as DERS-16, AAQ-II, and MPFI-EA for transdiagnostic factors and DASS-21 for stress) as five dependent variables.

MANCOVAs will also be performed to compare the mean levels of relevant unhealthy behavior (FTND, AUDIT, or SCOFF and EDE-Q scores), transdiagnostic factors (DERS-16, AAQ-II, and MPFI-EA scores), and stress levels (DASS-21-stress scale scores) of the experimental and control (waiting-list) groups of the samples at each assessment time (T1, T2, and T3), adjusting for any differences at T0 and age. In particular, three MANCOVAs will be run (one for T1 scores, one for T2 scores, and one for T3 scores) for each sample relative to the dysfunctional behaviors using group (VR or waiting-list) as an independent variable, questionnaire scores (one between FTND, AUDIT, or EDE-Q scores depending on the sample, the questionnaires DERS-16, AAQ-II, and MPFI-EA for transdiagnostic factors and the DASS-21 for stress) as five dependent variables, and scores at T0 and age as covariates.

All analyses will be performed following an Intention-To-Treat (ITT) approach in order to preserve the randomized design and provide a more conservative estimate of treatment effects.

## 3. Discussion and Clinical Implications

Unhealthy lifestyles and behaviors such as heavy drinking, smoking, and engaging in dysfunctional eating behaviors (e.g., bingeing and excessive dieting) are highly associated with health-related issues and risks [57,58,59]. Because of this, and also due to the observed increase in these unhealthy lifestyles and behaviors following the COVID-19 pandemic [60,61,62], improving the accessibility and reach of current psychological interventions to reduce smoking, alcohol use, and dysfunctional eating behaviors is of particular clinical relevance.

According to the literature, VR is a promising instrument to reduce these behaviors in several studies [5,6,25,26,27,28,29,30]. However, the disorder-specific approach that has been used for the development of VR software and interventions (with the exception of those developed to improve emotion regulation strategies [8]) has resulted in high implementation costs for their use in clinical psychology and has limited their application [63].

Applying a transdiagnostic approach to the development of VR software could help to overcome this issue. In fact, due to its ability to overcome some of the flaws concerning disorder-specific treatments (such as higher costs in terms of time and training and the need for multiple protocols aimed at tackling specific psychiatric diagnoses), the transdiagnostic approach has already gained support in the field of clinical psychology [64,65]. Since the H.O.M.E. software and the related VR-based intervention were created to be able to tackle several unhealthy lifestyles and behaviors (e.g., heavy drinking, smoking, and engaging in dysfunctional eating behaviors) together with several transdiagnostic factors linked to their maintenance (i.e., psychological flexibility, emotion regulation, and experiential avoidance), these instruments could be useful tools for clinicians to help more individuals to improve their health and well-being. Moreover, given the similarities between VR and technologies used in everyday life (e.g., smartphones and videogames), the tested VR-based intervention may help engage individuals who are reluctant towards traditional psychological prevention strategies, especially young people. In conclusion, if the expected beneficial effect of the H.O.M.E. protocol is confirmed after this research protocol, a new instrument to address psychological distress, unhealthy behaviors, and transdiagnostic psychological risk factors will be available, which could provide major advancements in the promotion of well-being in adults and young individuals [66,67].

## Data Availability

Due to the non-empirical nature of the work (protocol), no original data were collected for this article.
